# Aggressive end-of-life care among over half a million patients with cancer in Shandong, China, 2017–2022

**DOI:** 10.7189/jogh.16.04258

**Published:** 2026-07-31

**Authors:** Ying Meng, Qing Wang, Yu He, Yaoyun Zhang, Xinxin Xia, Xiaokang Ji, Qingbo Zhao, Yongchao Wang, Yifu Zhao, Ding Wang, Yang Ke, Fuzhong Xue, Jin Xu

**Affiliations:** 1 School of Public Health, Peking University, Beijing, China; 2 China Center for Health Development Studies, Peking University, Beijing, China; 3 School of Public Health, Shandong University, Jinan, China; 4 Chinese Preventive Medicine Association, Beijing, China; 5 State Key Laboratory of Molecular Oncology, Beijing Key Laboratory of Carcinogenesis and Translational Research, Department of Genetics, Peking University Cancer Hospital and Institute, Beijing, China; 6 World Health Organization Collaborating Centre for Universal Health Coverage, Beijing, China; 7 National Health Commission Key Laboratory of Health System Reform and Governance Research, Peking University, Beijing, China

**Keywords:** End-of-Life, aggressive cancer care, palliative care, anticancer treatment, low- and middle-income countries

## Abstract

**Background:**

Aggressive cancer care near the end of life (EoL) often yields limited benefits and may even diminish patients' quality of life. Research on EoL cancer care from low- and middle-income countries (LMICs) remains scarce. As China experiences accelerated population ageing, a large number of cancer deaths, and rapid expansion of hospital capacity and medical technologies, it is important to assess the trends and determinants of aggressive EoL care among patients with cancer.

**Methods:**

This cross-sectional study used linked hospital discharge and death registry data, including 594,415 adult patients who died of cancer between 1 January 2017, and 31 December 2022 in China. Aggressive EoL cancer care was measured using several internationally validated indicators, including chemotherapy use, multiple hospitalisations, and intensive care unit (ICU) admissions near the EoL. A modified Poisson regression model was applied to identify associated factors.

**Results:**

Among patients who died of cancer, 20.7% experienced at least one type of aggressive end-of-life care. From 2017 to 2022, the proportion of EoL ICU admissions increased from 0.9% to 2.8%, aggressive chemotherapy use rose from 4.1% to 4.8%, and multiple hospitalisations increased from 16.2% to 18.3%. Male sex, younger age, stable income, social health insurance with higher reimbursement rates, and comorbidities were factors significantly associated with a higher risk.

**Conclusions:**

This study offers a comprehensive analysis of aggressive EoL cancer care in an LMIC setting, revealing rising trends and key predictors. The findings underscore the urgent need to prioritise people-centred EoL care in rapidly evolving LMIC healthcare systems, while minimising unnecessary hospital-based interventions.

As China enters the stage of rapid population ageing, deaths are increasingly surpassing births, making end of life care (EoL) a growing policy priority. Indeed, China ranked 53rd out of 81 countries on the 2021 Quality of Death and Dying Index [1]. With about 2.6 million deaths in 2022 [2], cancer accounts for roughly one quarter of all deaths in China [3]. Therefore, EoL care for patients with cancer is a critical component of quality of death in China, particularly as cancer care activities and spending are largely concentrated in the advanced and terminal stages [4]. However, aggressive EoL care, commonly reflected by chemotherapy, repeated hospitalisations, and ICU admission near death, often provides limited therapeutic benefit, increases treatment burden and healthcare costs, and may reduce patients’ quality of life [5–7]. Moreover, the rapid expansion of hospital capacity and medical technologies, combined with limited availability of palliative care, may further increase the risk of aggressive, hospital-based care for patients with cancer at the EoL in China.

In high-income countries, aggressive EoL cancer care has long been recognised as a common concern, with research using validated indicators consistently showing that a considerable proportion of patients with cancer experience such care. For example, in Ontario, Canada, 22.4% of patients experienced at least one indicator of aggressive EoL care between 1993 and 2004 [8], and among decedents aged ≥65 years across seven developed nations in 2010, the proportion with EoL ICU admission ranged from 27.2% in the United States to 3.5% in Germany [9]. Based on previous evidence, palliative care has been shown to mitigate aggressive EoL cancer care and improve the quality of dying [10]. However, only about 14% of people worldwide who need palliative care receive it, and services remain heavily concentrated in high-income countries [11]. This suggests that patients in high-income settings may have greater opportunities to achieve better EoL quality – an observation that aligns with cross-country rankings in Quality Index of Death [12]. Previous research has also identified several other determinants of aggressive EoL cancer care, including patient demographics, socioeconomic status, cancer type, and timing of death [8,10,13]. Evidence from low- and middle-income countries (LMICs), however, remains limited.

Studies in China have documented substantial EoL medical spending and frequent financial hardship among patients with cancer [14], with higher spending closely linked to greater treatment intensity [15]. In response to growing concerns about EoL cancer care, China has increasingly promoted the development of palliative and hospice care. Since 2017, the government has launched pilot programmes in selected cities [16], and in 2024 issued guidelines to include these services in the health insurance fee schedule [17]. However, palliative and hospice care services remain mainly delivered through hospital-based units or designated beds [18], and both service provision and actual utilisation are still severely limited [17]. Public understanding of palliative and hospice care also remains low [19].

Although evidence on aggressive EoL cancer care in China has emerged [15,20], the existing studies are based on relatively small samples and rely on nonstandardised indicators. One study reported that 5.3% of patients (n = 3350) received chemotherapy within 14 days before death [21], another found that 5.9% and 49.7% of patients (n = 894) had an ICU or emergency department admission at the EoL [15], and a third documented that 47.9% of patients (n = 792) underwent aggressive life-extending treatments [22]. The lack of large, population-based studies on EoL cancer care limits understanding of the overall patterns, creating a critical knowledge gap that hampers timely policy action in China and similar settings. In response, we conducted a population-based study on aggressive EoL care among patients with cancer from Shandong, China, with a population of about 100 million. The study linked hospital discharge and death registration data from 2017 to 2022, assessed the aggressiveness of care, and examined the factors associated with aggressive care.

## METHODS

### Data and study population

The data for this study were obtained from the Cheeloo Lifespan Electronic Health Research Data-library (Cheeloo LEAD), a linked electronic health data platform built by the National Institute of Health Data Science of China (http://www.mhdata.sdu.edu.cn/). It systematically collects and archives hospital discharge data, specifically the standardised Hospitalisation Record Front Pages (HRFPs), from all secondary and tertiary hospitals in Shandong Province since 2009. The HRFPs summarise a range of information, including patient demographics as well as the diagnoses and treatments for each individual hospital admission. The HRFPs do not identify ward type, palliative care wards, or designated hospice beds; therefore, such admissions, if present, could not be distinguished from other hospital admissions. The data platform also incorporates death registry data from all of Shandong, which includes cause of death coded with International Classification of Diseases, 10th Revision (ICD-10) and date of death. While data on the platform were anonymised, each individual was assigned a unique encrypted identity number.

**Figure 1:**
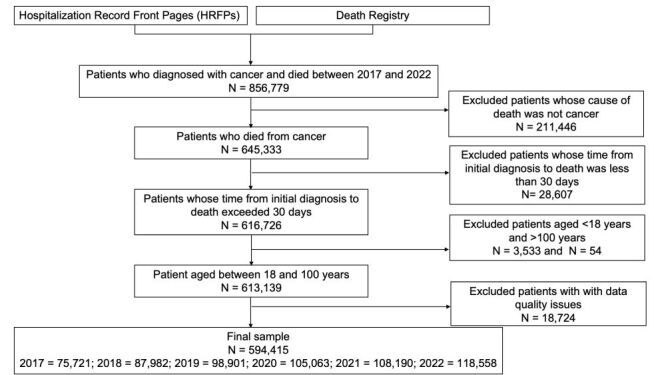
Sample derivation.

We first identified patients who were diagnosed with cancer and died between 1 January 2017 and 31 December 2022, through the linkage of HRFPs and death registry data, yielding a total of 856,779 individuals. We then excluded patients whose underlying cause of death was not cancer (n = 211,446). We also excluded patients who died within 30 days of initial diagnosis (n = 28,607), as very late diagnosis may have resulted in treatment decisions and care trajectories that differed from those of most patients with a longer disease course. Individuals younger than 18 years or older than 100 years were excluded (n = 3,587). Finally, we excluded patients with data quality issues (n = 18,724), primarily due to missing demographic or administrative variables that could not be supplemented from other available fields. These excluded patients were not concentrated in specific cities or hospitals (**Figure 1**).

### Variables

Aggressive EoL care was measured using a set of binary indicators derived from widely used quality indicators for end-of-life cancer care. These indicators were originally developed by Earle and colleagues through literature review, interviews with patients and bereaved family members, and expert assessment, and were subsequently operationalised and evaluated using administrative data [23,24]. In this study, aggressive EoL care was assessed using three indicators: chemotherapy administered within the last 14 days of life, multiple hospitalisations, defined as more than one hospitalisation during the final 30 days of life, and ICU admission during the final 30 days of life. A composite indicator of aggressive care was constructed and coded as 1 if a patient experienced any of these three indicators and 0 otherwise. Because HRFPs do not identify ward type, palliative care wards, or designated hospice beds, such admissions, if present, could not be distinguished from other hospital admissions.

Admission-level records were aggregated into patient-level indicators using each patient’s unique encrypted identity number. After removing duplicate records, all hospitalisations were sorted by admission date, and the date of death was used as the anchor to identify EoL care indicators from pre-death hospitalisation records. Multiple hospitalisations were defined as two or more distinct HRFP records overlapping the final 30-day window before death. Transfers generated new HRFP records and were counted as separate hospitalisations. No overlapping hospitalisation intervals were identified in the final analytic data set. For the chemotherapy indicator, chemotherapy was identified using the ICD code Z51.1 in hospitalisation records within the last 14 days of life. To reduce potential misclassification of palliative treatment as aggressive care, records specifically coded as palliative chemotherapy, Z51.104, were excluded from this indicator.

This study also examined two additional commonly used indicators, namely more than 14 inpatient days in the last 30 days of life and death in an acute care hospital. Both indicators primarily reflected inpatient utilisation and showed trends similar to those observed for multiple hospital admissions (Table S1 in the **Online Supplementary Document**). It should be noted that these two indicators were not included in the composite aggressive care variable.

Based on the previous literature and the availability of data, a range of factors potentially associated with aggressive EoL cancer care was examined. These included:

• patient demographics, *i.e.* sex, age at death, marital status, and ethnicity;

• socioeconomic factors, including employment status and city of residence, which captured a broad range of regional characteristics, such as economic conditions, health resource availability, and cultural differences across cities;

• noncancer comorbidities during the last six months of life using the Charlson Comorbidity Index (CCI) [25,26], a weighted index that can be scored from ICD-code data [25] (Table S2 in the **Online Supplementary Document**);

• cancer types, *i.e.* any one of the top ten solid tumours by mortality rate, based on death registry and ICD-code data (Table S3 in the **Online Supplementary Document**);

• year of death;

• type of insurance coverage, *i.e.* the Urban and Rural Resident Basic Medical Insurance (URRBMI) with lower reimbursement rate, the Urban Employee Basic Medical Insurance (UEBMI) with higher reimbursement rate, otherwise insured, or uninsured;

• level of hospital, *i.e.* secondary or tertiary, for the last hospitalisation before death.

### Statistical analysis

We used descriptive statistics to summarise patient characteristics overall and stratified by whether patients experienced any aggressive EoL cancer care. Pearson χ^2^ test was used to examine the relationships between aggressive EoL cancer care indicators and their influencing factors. We then used modified Poisson regression with robust error variance to estimate associations between the covariates listed above and aggressive EoL cancer care. The results are presented as relative risks (RRs) and associated 95% confidence intervals (CIs). All analyses were conducted using *R*, version 4.4.1 (R Foundation for Statistical Computing, Vienna, Austria).

To further characterise the diagnoses and procedures associated with ICU admissions and multiple admissions in the last 30 days, we conducted descriptive analyses of primary diagnosis and procedure codes in the corresponding hospitalisation records.

We performed a sensitivity analysis restricted to patients with metastatic cancer, identified using ICD-10 codes C77 to C80 from hospitalisation records in the year preceding death. Metastatic disease is commonly used as a marker of advanced cancer status [27,28], and this analysis examined whether the main findings were consistent in this subgroup. In addition, to assess the potential influence of COVID-19-related disruptions on temporal trends and associated factors, we stratified the modified Poisson regression models into the pre-pandemic period (2017–2019) and the pandemic period (2020–2022).

## RESULTS

### Patient characteristics

Among the 594,415 patients who died from cancer between 2017 and 2022, approximately half died at ages 61 to 75 years. Most patients were male (65.9%), farmers (53.0%), or covered by URRBMI (66.1%). 55.1% of the patients had their last hospitalisation in tertiary hospitals before they died. 50.3% of all patients had no non-cancer comorbidity, while 21.8% had a CCI score of 1, and 27.9% had a CCI score of 2 or above. Lung cancer was the most common cause of death (32.4%), followed by stomach cancer (13.9%) and liver cancer (12.6%) (**Table 1**). Results for marital status, ethnicity, patient residence, and year of death are provided in Table S4 in the **Online Supplementary Document**.

**Table 1:** Characteristics of patients by aggressive care, n (%)

Characteristic	All patients (n = 594,415)	Patients not experiencing any aggressive care (n = 471,478)	Patients experiencing any aggressive care (n = 122,937)	
**Sex**				
Female	202,847 (34.1)	163,998 (34.8)	38,849 (31.6)	
Male	391,568 (65.9)	307,480 (65.2)	84,088 (68.4)	
**Age group, years**				
18–45	23,939 (4.0)	16,297 (3.5)	7,642 (6.2)	
46–60	129,633 (21.8)	96,065 (20.4)	33,568 (27.3)	
61–75	292,713 (49.2)	234,223 (49.7)	58,490 (47.6)	
>75	148,130 (24.9)	124,893 (26.5)	23,237 (18.9)	
**Occupation and employment status**				
Farmers	315,136 (53.0)	260,943 (55.3)	54,193 (44.1)	
Public servants and retirees*	67,183 (11.3)	45,319 (9.6)	21,864 (17.8)	
Corporate employees	21,942 (3.7)	15,670 (3.3)	6,272 (5.1)	
Occupation without a stable source of income	20,912 (3.5)	16,101 (3.4)	4,811 (3.9)	
Others	169,242 (28.5)	133,445 (28.3)	35,797 (29.1)	
**Insurance**				
URRBMI	392,688 (66.1)	324,440 (68.8)	68,248 (55.5)	
UEBMI	140,409 (23.6)	98,150 (20.8)	42,259 (34.4)	
Uninsured	21,534 (3.6)	16,880 (3.6)	4,654 (3.8)	
Otherwise insured	39,784 (6.7)	32,008 (6.8)	7,776 (6.3)	
**Hospital**				
Secondary	267,104(44.9)	211,113 (44.8)	55,991 (45.5)	
Tertiary	327,311 (55.1)	260,365 (55.2)	66,946 (54.5)	
**Non-cancer CCI**				
0	299,137 (50.3)	261,147 (55.4)	37,990 (30.9)	
1	129,329 (21.8)	98,030 (20.8)	31,299 (25.5)	
≥2	165,949 (27.9)	112,301 (23.8)	53,648 (43.6)	
**Cancer type**				
Brain	12,779 (2.1)	11,065 (2.3)	1,714 (1.4)	
Breast	17,196 (2.9)	13,551 (2.9)	3,645 (3.0)	
Cervical	7,310 (1.2)	5,936 (1.3)	1,374 (1.1)	
Colorectal	39,749 (6.7)	32,050 (6.8)	7,699 (6.3)	
Oesophageal	49,531 (8.3)	40,257 (8.5)	9,274 (7.5)	
Liver	74,618 (12.6)	58,421 (12.4)	16,197 (13.2)	
Lung	192,449 (32.4)	154,290 (32.7)	38,159 (31.0)	
Pancreatic	19,951 (3.4)	15,283 (3.2)	4,668 (3.8)	
Prostate	5,785 (1.0)	4,833 (1.0)	952 (0.8)	
Stomach	82,517 (13.9)	66,482 (14.1)	16,035 (13.0)	
Others	92,530 (15.6)	69,310 (14.7)	23,220 (18.9)	
**Aggressive care**			
Chemotherapy in the last 14 days	25,943 (4.4)	-	-	
Multiple admissions in the last 30 days	104,023 (17.5)	-	-	
ICU in the last 30 days	11,294 (1.9)	-	-	
Any one of the above	122,937 (20.7)	-	-	

CCI – Charlson Comorbidity Index, EoL – end-of-life, ICU – intensive care unit, UEBMI – Urban Employee Basic Medical Insurance, URRBMI – Urban and Rural Resident Basic Medical Insurance

*Public servants and retirees include both public servants and retirees from all occupations. These two groups were combined because their occupation and employment status are generally associated with stable but relatively modest incomes in China.

Overall, 20.7% of patients received at least one type of aggressive care. This composite outcome was mainly driven by the relatively high prevalence of multiple hospitalisations within the last 30 days of life (17.5%), compared with chemotherapy in the last 14 days (4.4%) and ICU admission in the last 30 days (1.9%). The observed differences in characteristics across patients who did and did not receive aggressive care were statistically significant (**Table 1**). Descriptive analyses of primary diagnosis codes showed that diagnoses for ICU admissions and multiple admissions in the last 30 days were heterogeneous, with some leading codes related to supportive care, chemotherapy-related care, or malignancy itself. The most common codes are provided in Table S5 in the **Online Supplementary Document**.

### Trends in aggressive EoL care

**Figure 2:**
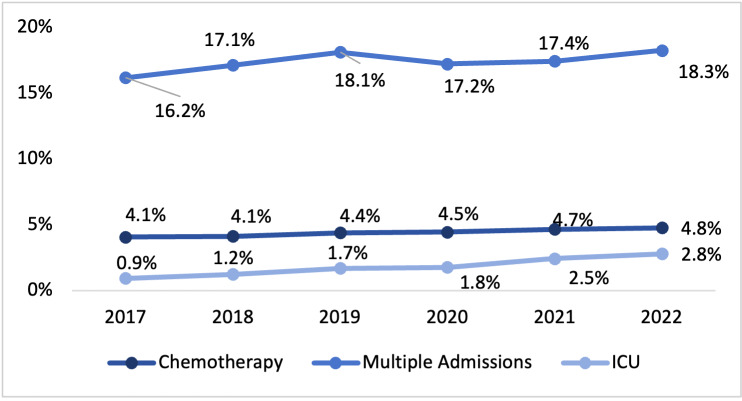
Proportions of EoL patients with cancer experiencing any indicator of aggressive care.

From 2017 to 2022, the absolute proportions of patients who experienced aggressive EoL cancer care increased across all indicators. The proportion of patients admitted to the ICU in the last 30 days of life rose from 0.9% to 2.8%, representing a substantial relative increase, although the absolute level remained low. Chemotherapy use increased from 4.1% to 4.8%, and multiple hospitalisations increased from 16.2% to 18.3% (**Figure 2**). Similar patterns were observed when trends were normalised using 2017 as the reference year (Figure S1 in the **Online Supplementary Document**). The magnitude and pattern of change varied across cancer types (Figure S2 in the **Online Supplementary Document**).

### Predictors of aggressive care

In the modified Poisson regression, female sex was associated with a slightly reduced chance of experiencing an indicator of aggressive care (RR = 0.97; 95% CI = 0.96–0.98). Compared to patients aged 18–45, the risk ratio of aggressive care was lower among those aged 46–60 (RR = 0.77; 95% CI = 0.76–0.79), 61–75 (RR = 0.56; 95% CI = 0.55–0.57), and over 75 (RR = 0.4; 95% CI = 0.39–0.40). Public servants and retirees (RR = 1.44; 95% CI = 1.42–1.46) had a higher risk ratio compared to farmers, while coverage by the UEBMI (RR = 1.35; 95% CI = 1.33–1.37) was associated with a higher risk compared to URRBMI. The absence of comorbidities was associated with decreased risk ratio of aggressive care (RR = 0.4; 95% CI = 0.40–0.41) (**Table 2**).

**Table 2:** Modified poisson regression analysis predicting aggressive care (95% CI)

Characteristics		Chemotherapy	Multiple Admissions	ICU	Any
**Sex (ref: male)**	Female	0.94 (0.91–0.96) *	0.98 (0.96–0.99) *	0.96 (0.92–1.00)	0.97 (0.96–0.98) *
**Age group (ref: 18-45)**	46–60	0.76 (0.72-0.80) *	0.76 (0.74-0.78) *	0.67 (0.62-0.73) *	0.77 (0.75–0.79) *
	61–75	0.54 (0.52–0.57) *	0.54 (0.53–0.56) *	0.57 (0.53–0.62) *	0.56 (0.55–0.57) *
	>75	0.22 (0.21–0.24) *	0.39 (0.38–0.40) *	0.49 (0.45–0.53) *	0.40 (0.39–0.40) *
**Occupation and employment status (ref: farmers)**	Public servants and retirees	1.10 (1.05–1.15) *	1.52 (1.49–1.55) *	1.49 (1.40–1.59) *	1.44 (1.42–1.46) *
	Corporate employees	1.00 (0.95–1.07)	1.27 (1.24–1.30) *	1.10 (0.99–1.21)	1.21 (1.18–1.24) *
	Occupation without a stable source of income	0.92 (0.87–0.98) †	1.23 (1.20–1.27) *	1.15 (1.04–1.28) ‡	1.17 (1.14–1.20) *
	Others	0.88 (0.85–0.91) *	1.12 (1.11–1.14) *	1.13 (1.08–1.18) *	1.08 (1.06–1.09) *
**Insurance type (ref: URRBMI)**	UEBMI	1.26 (1.22–1.31) *	1.37 (1.35–1.39) *	1.44 (1.37–1.52) *	1.35 (1.33–1.37) *
	Uninsured	0.95 (0.89–1.02)	1.21 (1.18–1.24) *	1.75 (1.61–1.91) *	1.17 (1.14–1.20) *
	Otherwise insured	0.99 (0.94–1.04)	1.11 (1.08–1.13) *	1.06 (0.98–1.15)	1.08 (1.06–1.10) *
**Year of death**	Per year	1.03 (1.03–1.04) *	1.00 (0.99–1.00) *	1.18 (1.17–1.20) *	1.01 (1.00–1.01) *
**Hospital (ref: secondary hospital)**	Tertiary hospital	1.07 (1.04–1.10) *	0.81 (0.80–0.82) *	0.95 (0.91–0.98) ‡	0.84 (0.84–0.85) *
**Cancer type, (ref: liver)**	Brain	0.99 (0.87–1.12)	0.70 (0.67–0.74)	2.46 (2.18–2.77) *	0.79 (0.76–0.82) *
	Breast	2.54 (2.35–2.74) *	1.09 (1.06–1.13) *	1.59 (1.39–1.81) *	1.19 (1.15–1.22) *
	Cervical	2.10 (1.87–2.35) *	1.00 (0.95–1.06)	1.70 (1.41–2.05) *	1.11 (1.05–1.16) *
	Colorectal	1.87 (1.75–2.01) *	1.12 (1.09–1.15) *	1.78 (1.63–1.96) *	1.17 (1.14–1.20) *
	Oesophageal	2.16 (2.02–2.30) *	1.19 (1.16–1.22) *	1.99 (1.82–2.16) *	1.25 (1.22–1.28) *
	Lung	2.24 (2.13–2.36) *	1.12 (1.10–1.14) *	1.86 (1.74–1.99) *	1.20 (1.18–1.22) *
	Pancreatic	1.60 (1.47–1.74) *	1.22 (1.18–1.25) *	0.99 (0.87–1.14)	1.23 (1.19–1.26) *
	Prostate	1.07 (0.88–1.31)	0.99 (0.93–1.05)	1.72 (1.42–2.08) *	1.04 (0.98–1.10)
	Stomach	2.07 (1.96–2.19) *	1.16 (1.14–1.19) *	1.36 (1.25–1.47) *	1.21 (1.19–1.23) *
	Other	3.29 (3.12–3.46) *	1.33 (1.30–1.35) ‡	2.02 (1.88–2.17) *	1.44 (1.41–1.46) *
**Non-cancer CCI, (ref:≥2)**	0	0.65 (0.63–0.67) *	0.37 (0.37–0.38) *	0.22 (0.21–0.23) *	0.40 (0.40–0.41) *
	1	1.04 (1.01–1.07) †	0.73 (0.72–0.74) *	0.62 (0.59–0.65) *	0.76 (0.75–0.77) *

CI – confidence interval, ICU – intensive care unit, RR – risk ratio, UEBMI – Urban Employee Basic Medical Insurance, URRBMI – Urban and Rural Resident Basic Medical Insurance.

*P < 0.01.

†*P* < 0.10.

‡*P* < 0.05.

Regression analyses indicated that year of death was an independent predictor of temporal changes in aggressive EoL care after adjustment for other covariates. Among the individual indicators, aggressive ICU admissions exhibited the strongest temporal increase (RR per year = 1.18; 95% CI = 1.17–1.20). Patients whose final hospitalisation occurred in tertiary hospitals had a higher risk of aggressive chemotherapy (RR = 1.07; 95% CI = 1.04–1.10) but lower risks of multiple admissions (RR = 0.81; 95% CI = 0.80–0.82) and ICU admissions (RR = 0.95; 95% CI = 0.91–0.98). Cancer type was a significant determinant of risk ratio of aggressive care. Among the top ten causes of cancer-specific deaths, breast cancer, pancreatic cancer, brain cancer and esophageal cancer, were the types with highest chances of receiving EoL chemotherapy (RR = 2.54; 95% CI = 2.35–2.74), multiple admissions (RR = 1.22; 95% CI = 1.18–1.25), ICU admissions (RR = 2.46; 95% CI = 2.18–2.77), and at least one of the three main indicators of aggressive care (RR = 1.25; 95% CI = 1.22–1.28), respectively.

Sensitivity analysis using the modified Poisson regression in the metastatic cancer subgroup yielded consistent results (Table S6 and S7 in the **Online Supplementary Document**). This subgroup, comprising approximately half of the overall cohort, exhibited slightly higher rates of EoL chemotherapy (5.8%) and multiple hospital admissions (23%), while ICU admission rates were identical to those in the full sample. In addition, analyses stratified by period showed that the main associated factors were broadly consistent in 2017–2019 and 2020–2022. The temporal trend also remained evident in both periods, particularly for ICU admissions in the last 30 days, although the annual increase was stronger before the pandemic than during 2020–2022. Annual increases in chemotherapy and multiple admissions were modest and weaker during 2020–2022 (Table S8 and S9 in the **Online Supplementary Document**).

## DISCUSSION

To our knowledge, this is the first population-based study of aggressive EoL cancer care in one of China’s most populous provinces. It addresses a major knowledge gap from LMICs, as most prior research has been limited to a few high-income countries [9,25,29]. Using linked hospital discharge and death registration data from Shandong Province covering over half a million cancer deaths (2017–2022), we found that a notable share of decedents received aggressive EoL care, with modest absolute increases over time. We also identified several demographics, socioeconomic, clinical, and system-related factors associated with its occurrence.

The most common form of aggressive care in our study by far was multiple hospital admissions in the last 30 days of life (17.5%), while 4.4% received chemotherapy in the last 14 days of life, and 1.9% were admitted to ICU in the last 30 days of life. The rate of multiple EoL hospitalisations in China was much higher than in the USA (<10.0% in 1999), Canada (7.5% in 2004) [8], and France (12.1% in 2010) [30]. This may partly reflect continued reliance on hospital-based EoL care in China, in the context of relatively high hospital bed availability, with 400–499 hospital beds per 100,000 population [31]. The proportion of patients with cancer receiving chemotherapy within the last 14 days of life in China exceeded that of Canada (2.9% in 2004) [8], but was lower than the USA (about 11.0% in 1999 [8] and 10.1–14.1% among patients under 65 years old from 2007 to 2014 [32], France (16.0% in 2010) and Jordan (8.3% from 2010 to 2012) [30,33]. EoL ICU admission rate in our study was lower than in high-income countries [9,32], likely constrained by the availability of ICU beds in China (4.75 per 100,000 population) compared with OECD countries (25.84 per 100,000 on average) [34].

Aggressive EoL cancer care in China appears to have increased over time. This pattern is broadly consistent with trends reported in the USA [13], Canada [8], and Jordan [33], but differs from Korea, where aggressive EoL care declined between 2012 and 2018 [35]. One possible explanation is that Korea expanded its hospice infrastructure during this period, alongside increasing awareness and use of hospice services [35]. The upward trend in aggressive EoL care during the study period may reflect the combined influence of supply-side, demand-side, and system-level factors. On the supply side, expanding hospital capacity may have increased the availability of aggressive care near death. Continued ICU bed expansion may have provided the capacity for more patients to receive intensive care near the EoL, offering a plausible supply-side context for the near-tripling of EoL ICU admissions between 2017 and 2022, although our data cannot directly link bed expansion to individual ICU admissions.

On the demand side, demographic and clinical characteristics, financial accessibility, and family preferences may also shape the use of aggressive EoL care. In line with prior research, we found female sex to be associated with lower risks of aggressive EoL care [15], while patients with comorbidities faced an increased risk [29], Previous studies have reported inconsistent findings regarding the association between age and aggressive cancer care [15,32]. Our results suggest that older age is associated with a lower likelihood of receiving aggressive EoL cancer care, possibly because younger patients or their families are more willing to take the chance and accept aggressive care with perceived life-extending potential. We also observed that having stable sources of income was associated with a higher likelihood of receiving aggressive treatment, in alignment with previous studies [36]. In addition, we found patients covered by social health insurance with higher reimbursement rates had an elevated risk. Finally, cultural norms rooted in Confucianism and filial piety may further reinforce family preferences for ‘doing everything possible’ to prolong life [37].

At the system level, aggressive EoL care may be reinforced by the limited availability of alternatives to hospital-based care. In addition, the fee schedule for EoL care remains underdeveloped. Several essential EoL services, such as psychological support, are not covered by China’s social health insurance schemes [38]. Beyond gaps in coverage, the current payment system provides weak incentives for their provision, due to low reimbursement rates, high patient cost-sharing [38], and the absence of a dedicated payment system for hospice care [39].

These findings, however, require careful contextual interpretation in China. Although chemotherapy, ICU admission, and multiple hospital admissions near death are widely used as indicators of aggressive EoL cancer care, they should not be interpreted as definitive evidence of inappropriate care at the individual patient level. In particular, repeated hospitalisations near death may partly reflect structural constraints rather than solely aggressive treatment preferences or inappropriate clinical decision-making. Nevertheless, Chinese evidence links chemotherapy near the EoL with more invasive resuscitation, higher in-hospital death, and poorer survival outcomes [21], and also links aggressive EoL care with higher expenditure [15]. Together with evidence that some Chinese patients prefer home death [40], these findings suggest that the indicators remain meaningful proxies of high-intensity, hospital-based EoL care. They help identify patterns of care that may worsen quality of death, conflict with patient preferences, and increase unnecessary financial burden.

Therefore, the issue of aggressive EoL cancer care in China requires timely policy responses. First, it is important to further promote development of palliative and hospice care, as is supported by international experiences. A network of community- and home-based palliative care units is needed [41,42], along with palliative care training to address current workforce shortages and competency gaps in this discipline [18]. Second, early EoL discussions between doctors and patients in cases of poor prognoses, along with timely initiation of palliative care, should be encouraged to enhance the quality of EoL care, particularly for patient groups with high risks of aggressive care, such as younger male patients with stable income, coverage of insurance with higher reimbursement rate, and certain types of cancer [10,43,44]. In addition, patients’ EoL preferences should be systematically documented and shared across providers [12]. Promoting Advance Care Planning is also an effective approach to help patients avoid unwanted aggressive EoL care [45]. Third, establishing a systematic payment framework for palliative and hospice care that defines the scope of covered services and sets appropriate reimbursement levels would help improve service availability and quality [46]. Fourth, although cultural norms are difficult to change in the short term, public education on the harms of aggressive EoL care and promotion of the concept of ‘death with dignity’ are essential [32]. These efforts may help increase the use of advance care planning and reduce the sense of guilt among family members when foregoing aggressive interventions. Finally, monitoring the aggressiveness of EoL cancer care using validated indicators and routine data is feasible and needed at various levels (*i.e.* facility, regional, national, *etc.*), as demonstrated in our paper. Ideally, further studies and monitoring activities should include outpatient data.

### Limitations

This study has several limitations. First, the absence of outpatient data may lead to an underestimation of the incidence of EoL chemotherapy. Nevertheless, the impact should be minimal, as chemotherapy for EoL cancer patients in China predominantly occurs during hospitalisation, owing to the severity of their conditions at that stage. Second, our data set lacked information on cancer stage at diagnosis and disease progression. Without staging data, we could not fully distinguish potentially inappropriate aggressive care from clinically reasonable treatment in patients with uncertain prognosis or unexpected deterioration. This may have led to overestimation of aggressive EoL care. In addition, our data set lacks information on reasons for ICU admission, so some admissions unrelated to cancer may have inflated estimates of aggressive ICU use. However, because we restricted the sample to patients whose underlying cause of death was cancer, most ICU admissions near the EoL were likely cancer-related. Third, this study is based on hospital-based data from a single, albeit large, province in China, and therefore its result may not be directly generalisable to other regions in China. Other LMICs may face different circumstances regarding aggressive EoL care, so our findings should therefore be interpreted with caution. Fourth, the indicators used to measure aggressive EoL care were originally developed and validated mainly in high-income settings. Although they have been used in previous Chinese studies, their contextual validity and selected time thresholds have not been formally validated in China. Fifth, the study period included the COVID-19 pandemic, which may have affected hospital utilisation, ICU capacity, and access to services. Although the period-stratified sensitivity analysis showed broadly consistent trends before and during the pandemic, pandemic-related disruptions may still have influenced the magnitude and interpretation of temporal changes. Future studies with longer follow-up are needed to assess whether these trends persist.

## CONCLUSIONS

This study provides a comprehensive analysis of the aggressiveness of EoL cancer care in Shandong, China, from 2017 to 2022, examining temporal trends and predictors of aggressive care. Our findings reveal that a notable proportion of patients with cancer received aggressive EoL care, with modest increases in chemotherapy use during the last 14 days of life, multiple hospitalisations during the final 30 days, and ICU admissions during the final 30 days over the study period. Younger age, male sex, stable income, and health insurance with higher reimbursement rates were associated with an increased likelihood of receiving aggressive care. Timely policy responses to aggressive EoL cancer care in China should include expanding community- and home-based palliative care with adequate investment and flexible payment models, promoting early EoL discussions for high-risk patients, establishing appropriate payment mechanisms for palliative and hospice care, strengthening public education to encourage advance directives and ‘death with dignity’, and routinely monitoring care aggressiveness with validated indicators.

## Additional material


Online Supplementary Document

## Data Availability

**Data availability:** The data used in this study are not publicly available due to privacy restrictions and data use agreements.
