# Supplementary material for: Aggressive end-of-life care among over half a million patients with cancer in Shandong, China, 2017–2022
Source: J Glob Health. 2026 Jul 31;16:04258. doi: 10.7189/jogh.16.04258 (PMC13424745; doi:10.7189/jogh.16.04258)
Supplement: Online Supplementary Document [file jogh-16-04258-s001.pdf]

**Supplement to: Meng Y, Wang Q, He Y, Zhang Y, Xia X, Ji X, et al. Aggressive end-of-life care among over half a million patients with cancer in Shandong, China, 2017–2022. J Glob Health. 2026;16:04258.**

Table S1 Modified Poisson Regression Analysis Predicting Aggressive Care (95% CI) （Continued）

Table S2 ICD-10 codes for Charlson Comorbidity Index

Table S3 ICD-10 codes for Different Types of Cancer

Table S4 Characteristics of Patients by Aggressive Care* (Continued)

Table S5 Leading Diagnosis and Procedure Codes Among Patients with ICU Admission or Multiple Hospitalizations in the Last 30 Days of Life

Figure S1 Proportions of EoL Patients with Cancer Experiencing Any Indicator of Aggressive care (Normalized as Percentages of 2017 Levels)

Figure S2 Trends in Aggressive EoL Care by Year of Death by Cancer Type (%)

Table S6 Characteristics of Patients by Aggressive Care (Patients with Metastatic Cancer)

Table S7 Modified Poisson Regression Analysis Predicting Aggressive Care (95% CI) (Patients with Metastatic Cancer)

Table S8 Modified Poisson Regression Analysis Predicting Aggressive Care (95% CI) (2017-2019)

Table S9 Modified Poisson Regression Analysis Predicting Aggressive Care (95% CI) (2020-2022)

**Table S1 Modified Poisson Regression Analysis Predicting Aggressive Care (95% CI)（Continued）**

| Factors | | More than 14 Inpatient Hospital Days in the Last 30 Days | Death in Hospital |
|---|---|---|---|
| **Sex (ref: Male)** | Female | 1.05 (1.04 - 1.07)*** | 0.96 (0.92 - 1.00) |
| **Age Group (ref: 18-45)** | 46-60 | 0.78 (0.77 - 0.80)*** | 0.67 (0.62 - 0.73)*** |
| | 61-75 | 0.58 (0.57 - 0.59)*** | 0.57 (0.53 - 0.62)*** |
| | >75 | 0.47 (0.46 - 0.48)*** | 0.49 (0.45 - 0.53)*** |
| **Occupation and employment status (ref: Farmers)** | Public servants and retirees | 1.66 (1.63 - 1.68)*** | 1.49 (1.40 - 1.59)** |
| | Corporate employees | 1.41 (1.39 - 1.44)*** | 1.10 (1.00 - 1.21) |
| | Occupation without a stable source of income | 1.24 (1.21 - 1.27)*** | 1.15 (1.04 - 1.28)*** |
| | Others | 1.20 (1.19 - 1.22)*** | 1.13 (1.08 - 1.18)*** |
| **Insurance Type (ref: URRBMI)** | UEBMI | 1.55 (1.53 - 1.57)*** | 1.44 (1.37 - 1.52)*** |
| | Uninsured | 1.05 (1.02 - 1.07)*** | 1.75 (1.61 - 1.91)*** |
| | Otherwise insured | 1.19 (1.16 - 1.21)*** | 1.06 (0.98 - 1.15) |
| **Year of Death** | Per year | 0.95 (0.95 – 0.95)*** | 0.98 (0.98 – 0.98)*** |
| **Hospital (ref: Secondary Hospital)** | Tertiary Hospital | 0.76 (0.75 - 0.76)*** | 0.95 (0.91 - 0.98)*** |
| **Cancer type (ref: Lung)** | Brain | 0.88 (0.85 - 0.92)*** | 2.46 (2.18 - 2.77)*** |
| | Breast | 1.00 (0.97 - 1.03) | 1.59 (1.39 - 1.81)*** |
| | Cervical | 0.98 (0.93 - 1.02) | 1.70 (1.41 - 2.05)*** |
| | Colorectal | 1.11 (1.09 - 1.13)*** | 1.78 (1.63 - 1.96)*** |
| | Esophageal | 1.11 (1.09 - 1.13)*** | 1.99 (1.82 - 2.16)*** |
| | Lung | 1.07 (1.06 - 1.09)*** | 1.86 (1.74 - 1.99)*** |
| | Pancreatic | 1.31 (1.28 - 1.35)*** | 0.99 (0.87 - 1.14) |
| | Prostate | 0.95 (0.90 - 1.00)* | 1.72 (1.42 - 2.08)*** |
| | Stomach | 1.13 (1.11 - 1.15)*** | 1.36 (1.25 - 1.47)*** |
| | Other | 1.26 (1.24 - 1.28)*** | 2.02 (1.88 - 2.17)*** |
| **Non-cancer CCI** (ref: >=2)** | 0 | 0.46 (0.46 - 0.47)*** | 0.22 (0.21 - 0.24) |
| | 1 | 0.81 (0.80 - 0.82)*** | 0.62 (0.59 - 0.65)*** |

* p< 0.1, ** p< 0.05, *** p< 0.01

** Abbreviation: CCI=Charlson Comorbidity Index

**Table S2 ICD-10 codes for Charlson Comorbidity Index**

| ICD-10 Codes | Comorbid Condition | Weight |
|---|---|---|
| I21 | Myocardial infarction | 1 |
| I22 | | |
| I25.2 | | |
| I09.9 | Congestive heart failure | 1 |
| I11.0 | | |
| I13.0 | | |
| I13.2 | | |
| I25.5 | | |
| I42.0 | | |
| I42.5−I42.9 | | |
| I43 | | |
| I50 | | |
| P29.0 | | |
| I70 | Peripheral vascular disease | 1 |
| I71 | | |
| I73.1 | | |
| I73.8 | | |
| I73.9 | | |
| I77.1 | | |
| I79.0 | | |
| I79.2 | | |
| K55.1 | | |
| K55.8 | | |
| K55.9 | | |
| Z95.8 | | |
| Z95.9 | | |
| G45 | Cerebrovascular disease | 1 |
| G46 | | |
| I60−I69 | | |
| H34.0 | | |
| F00−F03 | Dementia | 1 |
| G30 | | |
| F05.1 | | |
| G31.1 | | |
| I27.8 | Chronic pulmonary disease | 1 |
| I27.9 | | |
| J40−J47 | | |
| J60−J67 | | |
| J68.4 | | |
| J70.1 | | |
| J70.3 | | |
| M05 | Rheumatologic disease | 1 |
| M06 | | |
| M31.5 | | |
| M32−M34 | | |
| M35.1 | | |
| M35.3 | | |
| M36.0 | | |
| B18 | Mild liver disease | 1 |
| K70.0−K70.3 | | |
| K70.9 | | |
| K71.3−K71.5 | | |
| K71.7 | | |

| | | |
|---|---|---|
| K73 | | |
| K74 | | |
| K76.0 | | |
| K76.2−K76.4 | | |
| K76.8 | | |
| K76.9 | | |
| Z94.4 | | |
| E10.0 | Diabetes without complications | 1 |
| E10.1 | | |
| E10.6 | | |
| E10.8 | | |
| E10.9 | | |
| E11.0 | | |
| E11.1 | | |
| E11.6 | | |
| E11.8 | | |
| E11.9 | | |
| E12.1 | | |
| E12.6 | | |
| E12.8 | | |
| E12.9 | | |
| E13.0 | | |
| E13.1 | | |
| E13.6 | | |
| E13.8 | | |
| E13.9 | | |
| E14.0 | | |
| E14.1 | | |
| E14.6 | | |
| E14.8 | | |
| E14.9 | | |
| G04.1 | Hemiplegia/ paraplegia | 2 |
| G11.4 | | |
| G80.1 | | |
| G80.2 | | |
| G81 | | |
| G82 | | |
| G83.0 | | |
| G83.1 | | |
| G83.2 | | |
| G83.3 | | |
| G83.4 | | |
| G83.9 | | |
| E10.2−DE10.5 | Diabetes with chronic complications | 2 |
| E10.7 | | |
| E11.2 | | |
| E11.5 | | |
| E11.7 | | |
| E12.2−E12.5 | | |
| E12.7 | | |
| E13.2−E13.5 | | |
| E13.7 | | |
| E14.2−E14.5 | | |
| E14.7 | | |
| I12.0 | Renal disease | 2 |
| I13.1 | | |
| N03.2−N03.7 | | |
| N05.2−N05.7 | | |

| | | |
|---|---|---|
| N18 | | |
| N19 | | |
| N25.0 | | |
| Z49.0−Z49.2 | | |
| Z94.0 | | |
| Z99.2 | | |
| I85.0 | Moderate/severe liver disease | 3 |
| I85.9 | | |
| I86.4 | | |
| I98.2 | | |
| K70.4 | | |
| K71.1 | | |
| K72.1 | | |
| K72.9 | | |
| K76.5−K76.7 | | |
| B20−B22 | HIV/AIDS | 6 |
| B24 | | |

**Table S3 ICD-10 codes for Different Types of Cancer**

| Cancer Type | ICD-10 Codes |
|---|---|
| Brain | C70-C72 |
| Breast | C50 |
| Cervical | C53 |
| Colorectal | C18-C21 |
| Esophageal | C15 |
| Liver | C22 |
| Lung | C33 |
| Pancreatic | C25 |
| Prostate | C61 |
| Stomach | C16 |

**Table S4. Characteristics of Patients by Aggressive Care (Continued)**

| Characteristic | All Patients (n=594,415) | Patients Not Experiencing Any Aggressive Care (n=471,478) | Patients Experiencing Any Aggressive Care (n=122,937) | P-value* |
|---|---|---|---|---|
| **Marital Status** | | | | |
| Married | 92.20% | 92.10% | 92.70% | P<0.001 |
| Widowed/divorced | 3.20% | 3.30% | 2.80% | |
| Unmarried | 2.80% | 2.80% | 2.70% | |
| Unknown | 1.80% | 1.80% | 1.80% | |
| **Ethnicity** | | | | |
| Han | 94.80% | 94.90% | 94.80% | P<0.001 |
| Hui | 0.30% | 0.30% | 0.30% | |
| Foreign nationality | 0.40% | 0.40% | 0.40% | |
| Unknown | 3.50% | 3.50% | 3.40% | |
| Others | 1.00% | 0.90% | 1.10% | |
| **City** | | | | |
| City A | 11.30% | 11.80% | 9.50% | P<0.001 |
| City B | 4.70% | 4.80% | 4.10% | |
| City C | 4.60% | 4.80% | 4.10% | |
| City D | 3.20% | 3.10% | 3.70% | |
| City E | 6.10% | 6.30% | 5.30% | |
| City F | 8.60% | 8.20% | 10.40% | |
| City G | 7.30% | 7.30% | 7.40% | |
| City H | 4.50% | 4.50% | 4.60% | |
| City I | 10.90% | 11.20% | 9.50% | |
| City J | 11.50% | 11.10% | 12.90% | |
| City K | 2.80% | 2.60% | 3.50% | |
| City L | 7.60% | 7.80% | 6.80% | |
| City M | 4.30% | 4.20% | 4.60% | |
| City N | 3.80% | 3.80% | 3.70% | |
| City O | 3.40% | 3.50% | 3.00% | |
| City P | 5.50% | 5.20% | 7.10% | |
| **Year of Death** | | | | |
| 2017 | 12.70% | 13.00% | 11.60% | |
| 2018 | 14.80% | 14.90% | 14.20% | |
| 2019 | 16.60% | 16.50% | 17.10% | |

| | | | | |
|---|---|---|---|---|
| 2020 | 17.70% | 17.70% | 17.40% | P<0.001 |
| 2021 | 18.20% | 18.10% | 18.50% | |
| 2022 | 19.90% | 19.60% | 21.10% | |
| **Aggressive Care** | | | | |
| > 14 Inpatient Hospital Days in the Last 30 Days | 22.91% | - | - | |
| Death in Hospital | 22.34% | - | - | |

*p-value < 0.05, statistically significant

**Table S5 Leading Diagnosis and Procedure Codes Among Patients with ICU Admission or Multiple Hospitalizations in the Last 30 Days of Life**

| | | | | |
|---|---|---|---|---|
| **Panel A. Diagnosis codes among patients with ICU admission in the last 30 days of life** | | | | |
| **Rank** | **ICD diagnosis code** | **No. of patients** | **Proportion** | **ICD diagnosis name** |
| 1 | Z51.103 | 624 | 1% | Maintenance chemotherapy for malignant neoplasm |
| 2 | Z51.100 | 566 | 1% | Encounter for chemotherapy for malignant neoplasm |
| 3 | Z51.102 | 328 | 0% | Postoperative chemotherapy for malignant neoplasm |
| 4 | Z51.003 | 221 | 0% | Radiotherapy for malignant neoplasm |
| 5 | C34.900x001 | 198 | 0% | Malignant neoplasm of bronchus or lung |
| 6 | Z51.500x002 | 177 | 0% | Palliative care |
| 7 | Z51.801 | 172 | 0% | Targeted therapy for malignant neoplasm |
| 8 | J18.903 | 170 | 0% | Severe pneumonia |
| 9 | J98.414 | 166 | 0% | Pulmonary infection |
| 10 | Z51.901 | 163 | 0% | Symptomatic treatment |
| | **Total** | **11,294** | | |
| **Panel B. Procedure codes among patients with ICU admission in the last 30 days of life** | | | | |
| **Rank** | **Procedure code** | **No. of patients** | **Proportion** | **Procedure name** |
| 1 | NA | 5,956 | 53% | No procedure recorded |
| 2 | 99.2503 | 682 | 6% | Intravenous administration of chemotherapeutic agent |
| 3 | 96.04 | 545 | 5% | Endotracheal intubation |
| 4 | 96.7101 | 167 | 1% | Mechanical ventilation for less than 96 hours |
| 5 | 96.7201 | 118 | 1% | Mechanical ventilation for 96 consecutive hours or more |
| 6 | 39.7903 | 114 | 1% | Transcatheter hepatic arterial embolization |
| 7 | 34.0401 | 107 | 1% | Closed thoracic drainage |
| 8 | 92.2400x003 | 92 | 1% | Intensity-modulated radiation therapy |
| 9 | 99.2800x006 | 81 | 1% | Injection or infusion of biological response modifier as an antineoplastic agent |
| 10 | 41.3800x001 | 76 | 1% | Bone marrow aspiration |
| | **Total** | **11,294** | | |
| **Panel C. Diagnosis codes among patients with multiple hospitalizations in the last 30 days of life** | | | | |

| Rank | ICD diagnosis code | No. of admissions | Proportion | ICD diagnosis name |
|---|---|---|---|---|
| 1 | Z51.500x002 | 6,642 | 6% | Supportive care for malignant neoplasm |
| 2 | Z51.901 | 4,794 | 5% | Symptomatic treatment |
| 3 | C22.900 | 3,821 | 4% | Malignant neoplasm of liver |
| 4 | C16.900 | 3,401 | 3% | Malignant neoplasm of stomach |
| 5 | C34.900x001 | 3,292 | 3% | Malignant neoplasm of lung |
| 6 | Z51.500x003 | 2,925 | 3% | Terminal maintenance treatment for malignant neoplasm |
| 7 | C34.900 | 2,574 | 2% | Malignant neoplasm of bronchus or lung |
| 8 | C15.900 | 2,326 | 2% | Malignant neoplasm of esophagus |
| 9 | C34.900x005 | 2,178 | 2% | Malignant neoplasm of right lung |
| 10 | Z51.103 | 1,594 | 2% | Maintenance chemotherapy for malignant neoplasm |
| | **Total** | **104,023** | | |

**Panel D. Procedure codes among patients with multiple hospitalizations in the last 30 days of life**

| Rank | Procedure code | No. of admissions | Proportion | Procedure name |
|---|---|---|---|---|
| 1 | NA | 54,438 | 52% | No procedure recorded |
| 2 | 96.04 | 2,714 | 3% | Endotracheal intubation |
| 3 | 54.9101 | 2,433 | 2% | Abdominal puncture and drainage |
| 4 | 99.2503 | 1,510 | 1% | Intravenous administration of chemotherapeutic agent |
| 5 | 34.0401 | 1,014 | 1% | Closed thoracic drainage |
| 6 | 54.9105 | 806 | 1% | Abdominal puncture |
| 7 | 38.9301 | 766 | 1% | Peripherally inserted central venous catheter placement |
| 8 | 96.7101 | 739 | 1% | Mechanical ventilation for less than 96 hours |
| 9 | 99.0401 | 635 | 1% | Red blood cell transfusion |
| 10 | 57.94 | 545 | 1% | Indwelling urinary catheter insertion |
| | **Total** | **104,023** | | |

**Figure S1 Proportions of EoL Patients with Cancer Experiencing Any Indicator of Aggressive Care (Normalized as Percentages of 2017 Levels)**

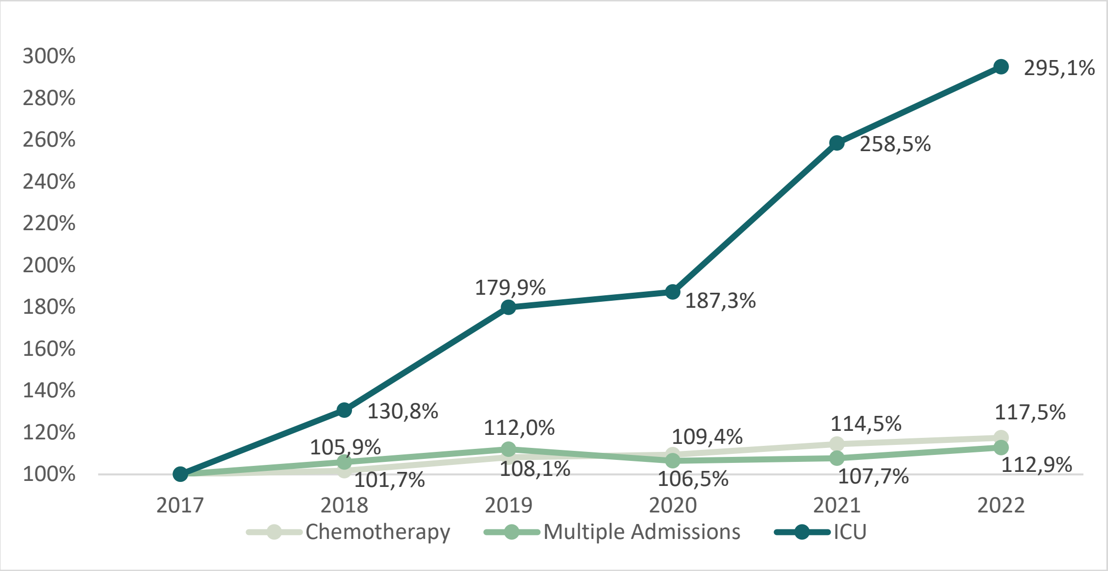

**Figure S2 Trends in Aggressive EoL Care by Year of Death by Cancer Type (%)**

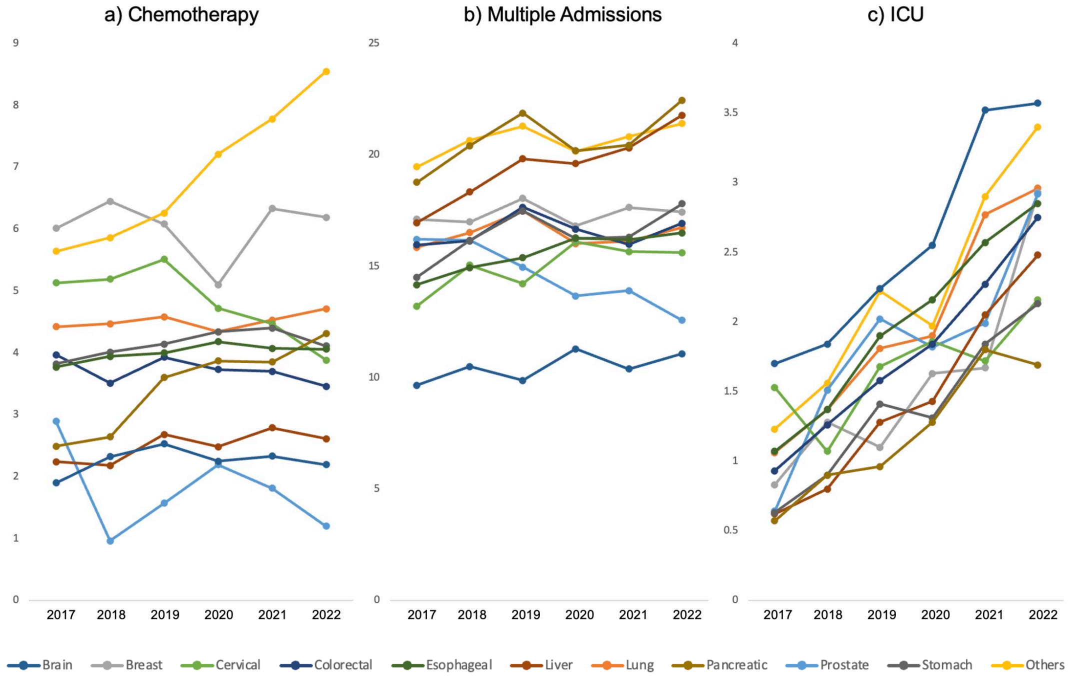

**Table S6 Modified Poisson Regression Analysis Predicting Aggressive Care (95% CI) (2017-2019)**

| Characteristics | | Chemotherapy | Multiple Admissions | ICU | Any |
|---|---|---|---|---|---|
| Sex (ref: Male) | Female | 0.942 (0.904 - 0.982)** | 0.975 (0.957 - 0.994)** | 0.958 (0.887 - 1.035) | 0.971 (0.955 - 0.988)*** |
| Age Group (ref: 18-45) | 46-60 | 0.788 (0.733 - 0.847)*** | 0.773 (0.747 - 0.8)*** | 0.663 (0.571 - 0.77)*** | 0.78 (0.756 - 0.804)*** |
| | 61-75 | 0.544 (0.506 - 0.584)*** | 0.572 (0.552 - 0.592)*** | 0.607 (0.526 - 0.7)*** | 0.582 (0.564 - 0.6)*** |
| | >75 | 0.222 (0.203 - 0.243)*** | 0.435 (0.419 - 0.452)*** | 0.525 (0.451 - 0.611)*** | 0.427 (0.413 - 0.442)*** |
| Occupation and employment status (ref: Farmers) | Public servants and retirees | 1.072 (1.001 - 1.148)* | 1.595 (1.552 - 1.639)*** | 1.656 (1.482 - 1.85)*** | 1.498 (1.461 - 1.536)*** |
| | Corporate employees | 1.088 (0.998 - 1.187) | 1.347 (1.296 - 1.4)*** | 1.243 (1.055 - 1.465)** | 1.293 (1.249 - 1.339)*** |
| | Occupation without a stable source of income | 0.946 (0.855 - 1.046) | 1.256 (1.202 - 1.312)*** | 0.984 (0.799 - 1.212) | 1.188 (1.141 - 1.237)*** |
| | Others | 0.87 (0.831 - 0.91)*** | 1.175 (1.15 - 1.2)*** | 1.153 (1.061 - 1.253)*** | 1.117 (1.096 - 1.138)*** |
| Insurance Type (ref: URRBMI) | UEBMI | 1.293 (1.231 - 1.359)*** | 1.381 (1.352 - 1.411)*** | 1.502 (1.375 - 1.64)*** | 1.369 (1.343 - 1.395)*** |
| | Uninsured | 1.001 (0.93 - 1.076) | 1.179 (1.142 - 1.216)*** | 1.091 (0.957 - 1.243) | 1.141 (1.108 - 1.174)*** |
| | Otherwise insured | 0.956 (0.869 - 1.052) | 1.225 (1.178 - 1.275)*** | 1.808 (1.575 - 2.077)*** | 1.185 (1.143 - 1.229)*** |
| Year of Death | Per year | 1.047 (1.023 - 1.071)*** | 1.039 (1.028 - 1.049)*** | 1.281 (1.227 - 1.337)*** | 1.043 (1.034 - 1.053)*** |
| Hospital (ref: Secondary Hospital) | Tertiary Hospital | 0.87 (0.837 - 0.905)*** | 0.825 (0.81 - 0.839)*** | 0.777 (0.726 - 0.832)** | 0.821 (0.809 - 0.835)*** |
| Cancer type (ref:Liver) | Brain | 1.037 (0.86 - 1.251) | 0.713 (0.658 - 0.773)*** | 2.913 (2.337 - 3.63)*** | 0.793 (0.738 - 0.852)*** |
| | Breast | 2.674 (2.38 - 3.004)*** | 1.167 (1.106 - 1.231)*** | 1.687 (1.314 - 2.166)*** | 1.274 (1.214 - 1.336)*** |
| | Cervical | 2.369 (2.012 - 2.789)*** | 1.015 (0.932 - 1.105) | 2.33 (1.712 - 3.171)*** | 1.163 (1.08 - 1.252)*** |
| | Colorectal | 1.999 (1.802 - 2.218)*** | 1.143 (1.098 - 1.189)*** | 1.945 (1.633 - 2.317)*** | 1.204 (1.161 - 1.249)*** |
| | Esophageal | 2.207 (2.007 - 2.429)*** | 1.197 (1.153 - 1.243)*** | 2.15 (1.84 - 2.511)*** | 1.261 (1.219 - 1.306)*** |
| | Lung | 2.328 (2.157 - 2.513)*** | 1.172 (1.141 - 1.204)*** | 2.037 (1.797 - 2.31)*** | 1.251 (1.22 - 1.282)*** |
| | Pancreatic | 1.412 (1.226 - 1.627)*** | 1.25 (1.193 - 1.311)*** | 1.076 (0.829 - 1.398) | 1.252 (1.198 - 1.309)*** |
| | Prostate | 1.121 (0.815 - 1.541) | 1.041 (0.947 - 1.145) | 1.928 (1.354 - 2.745)*** | 1.078 (0.986 - 1.178) |
| | Stomach | 2.105 (1.932 - 2.293)*** | 1.187 (1.15 - 1.225)*** | 1.52 (1.308 - 1.766)*** | 1.235 (1.2 - 1.272)*** |
| | Other | 2.92 (2.696 - 3.162)*** | 1.377 (1.337 - 1.417)*** | 2.427 (2.122 - 2.777)*** | 1.466 (1.427 - 1.505)*** |
| Non-cancer CCI* (ref:>=2) | 0 | 0.718 (0.686 - 0.752)*** | 0.403 (0.395 - 0.411)*** | 0.264 (0.243 - 0.287)*** | 0.436 (0.429 - 0.444)*** |
| | 1 | 1.048 (0.996 - 1.103) | 0.757 (0.742 - 0.773)*** | 0.659 (0.608 - 0.714)*** | 0.781 (0.767 - 0.796)*** |

* p< 0.1, ** p< 0.05, *** p< 0.01

Abbreviation: CCI=Charlson Comorbidity Index

**Table S7 Modified Poisson Regression Analysis Predicting Aggressive Care (95% CI) (2020-2022)**

| Characteristics | | Chemotherapy | Multiple Admissions | ICU | Any |
|---|---|---|---|---|---|
| **Sex (ref: Male)** | Female | 0.928 (0.896 - 0.961)*** | 0.975 (0.959 - 0.991)** | 0.962 (0.914 - 1.012) | 0.975 (0.96 - 0.989)*** |
| **Age Group (ref: 18-45)** | 46-60 | 0.734 (0.687 - 0.784)*** | 0.75 (0.727 - 0.774)*** | 0.67 (0.605 - 0.742)*** | 0.758 (0.737 - 0.779)*** |
| | 61-75 | 0.538 (0.504 - 0.574)*** | 0.52 (0.504 - 0.536)*** | 0.548 (0.496 - 0.605)*** | 0.54 (0.525 - 0.555)*** |
| | >75 | 0.226 (0.209 - 0.244)*** | 0.362 (0.35 - 0.374)*** | 0.471 (0.424 - 0.523)*** | 0.371 (0.36 - 0.382)*** |
| **Occupation and employment status (ref: Farmers)** | Public servants and retirees | 1.125 (1.062 - 1.192)*** | 1.495 (1.458 - 1.533)*** | 1.419 (1.312 - 1.534)*** | 1.422 (1.391 - 1.455)*** |
| | Corporate employees | 0.965 (0.889 - 1.048) | 1.221 (1.179 - 1.265)*** | 1.031 (0.912 - 1.166) | 1.16 (1.124 - 1.198)*** |
| | Occupation without a stable source of income | 0.924 (0.852 - 1.002) | 1.222 (1.178 - 1.268)*** | 1.207 (1.073 - 1.359)** | 1.172 (1.133 - 1.211)*** |
| | Others | 0.892 (0.856 - 0.93)*** | 1.076 (1.055 - 1.097)*** | 1.11 (1.048 - 1.176)*** | 1.046 (1.028 - 1.064)*** |
| **Insurance Type (ref: URRBMI)** | UEBMI | 1.23 (1.177 - 1.285)*** | 1.351 (1.324 - 1.378)*** | 1.416 (1.33 - 1.508)*** | 1.328 (1.304 - 1.352)*** |
| | Uninsured | 0.954 (0.888 - 1.025) | 1.05 (1.016 - 1.086)** | 1.054 (0.954 - 1.165) | 1.028 (0.998 - 1.059) |
| | Otherwise insured | 0.906 (0.819 - 1.002) | 1.199 (1.152 - 1.248)*** | 1.732 (1.552 - 1.932)*** | 1.157 (1.115 - 1.2)*** |
| **Year of Death** | Per year | 1.033 (1.014 - 1.053)*** | 1.01 (1.001 - 1.019)* | 1.204 (1.171 - 1.237)*** | 1.018 (1.01 - 1.026)*** |
| **Hospital (ref: Secondary Hospital)** | Tertiary Hospital | 1.247 (1.206 - 1.291)*** | 0.809 (0.797 - 0.822)*** | 1.03 (0.984 - 1.078) | 0.866 (0.854 - 0.878)*** |
| **Cancer type (ref:Liver)** | Brain | 0.955 (0.812 - 1.124) | 0.693 (0.649 - 0.741)*** | 2.301 (1.998 - 2.65)*** | 0.782 (0.738 - 0.828)*** |
| | Breast | 2.458 (2.217 - 2.726)*** | 1.039 (0.991 - 1.089) | 1.547 (1.323 - 1.809)*** | 1.12 (1.074 - 1.169)*** |
| | Cervical | 1.894 (1.616 - 2.221)*** | 0.992 (0.923 - 1.066) | 1.459 (1.154 - 1.844)** | 1.062 (0.996 - 1.133) |
| | Colorectal | 1.789 (1.635 - 1.957)*** | 1.092 (1.056 - 1.13)*** | 1.714 (1.538 - 1.91)*** | 1.135 (1.101 - 1.17)*** |
| | Esophageal | 2.112 (1.94 - 2.299)*** | 1.179 (1.141 - 1.219)*** | 1.915 (1.727 - 2.123)*** | 1.24 (1.204 - 1.278)*** |
| | Lung | 2.173 (2.033 - 2.324)*** | 1.078 (1.053 - 1.103)*** | 1.788 (1.653 - 1.935)*** | 1.154 (1.129 - 1.178)*** |
| | Pancreatic | 1.714 (1.54 - 1.906)*** | 1.182 (1.137 - 1.228)*** | 0.956 (0.817 - 1.118) | 1.195 (1.153 - 1.238)*** |
| | Prostate | 1.046 (0.808 - 1.353) | 0.948 (0.873 - 1.03) | 1.639 (1.307 - 2.055)*** | 1.002 (0.93 - 1.079) |
| | Stomach | 2.044 (1.896 - 2.202)*** | 1.135 (1.104 - 1.166)*** | 1.288 (1.168 - 1.42)*** | 1.183 (1.154 - 1.213)*** |
| | Other | 3.547 (3.316 - 3.794)*** | 1.287 (1.256 - 1.319)*** | 1.863 (1.71 - 2.03)*** | 1.409 (1.377 - 1.441)*** |
| **Non-cancer CCI* (ref:>=2)** | 0 | 0.595 (0.573 - 0.617)*** | 0.35 (0.344 - 0.356)*** | 0.205 (0.193 - 0.218)*** | 0.378 (0.372 - 0.384)*** |
| | 1 | 1.041 (1.002 - 1.082)* | 0.716 (0.703 - 0.728)*** | 0.604 (0.573 - 0.637)*** | 0.752 (0.74 - 0.764)*** |

* p< 0.1, ** p< 0.05, *** p< 0.01

Abbreviation: CCI=Charlson Comorbidity Index

**Table S8 Characteristics of Patients by Aggressive Care (Patients with Metastatic Cancer)**

| Characteristic | All Patients (n=278,086) | Patients Not Experiencing Any Aggressive Care (n=203,841) | Patients Experiencing Any Aggressive Care (n=74,245) | P-value |
|---|---|---|---|---|
| **Gender** | | | | |
| Female | 35.1% | 36.0% | 32.6% | <0.001 |
| Male | 64.9% | 64.0% | 67.4% | |
| **Age group** | | | | |
| 18-45 | 4.6% | 4.0% | 6.2% | <0.001 |
| 46-60 | 26.7% | 25.3% | 30.6% | |
| 61-75 | 51.1% | 52.1% | 48.6% | |
| >75 | 17.5% | 18.6% | 14.6% | |
| **Occupation and employment status** | | | | |
| Famers | 51.8% | 54.8% | 43.4% | <0.001 |
| Public servants and retirees | 13.0% | 10.9% | 18.7% | |
| Corporate employees | 4.2% | 3.8% | 5.4% | |
| Occupation without a stable source of income | 3.60% | 3.50% | 3.80% | |
| Others | 27.5% | 27.0% | 28.6% | |
| **Insurance** | | | | |
| URRBMI | 63.9% | 67.4% | 54.3% | <0.001 |
| UEBMI | 26.8% | 23.4% | 36.4% | |
| Uninsured | 3.4% | 3.3% | 3.5% | |
| Otherwise insured | 5.9% | 5.9% | 5.8% | |
| **Hospital** | | | | |
| Secondary | 43.4% | 42.9% | 44.7% | <0.001 |
| Tertiary | 56.6% | 57.1% | 55.3% | |
| **Non-cancer CCI** | | | | |
| 0 | 46.0% | 50.7% | 33.1% | <0.001 |
| 1 | 24.8% | 24.2% | 26.5% | |
| ≥ 2 | 29.2% | 25.2% | 40.4% | |
| **Cancer Type** | | | | |

| | | | | |
|---|---|---|---|---|
| Brain | 1.0% | 1.1% | 0.7% | |
| Breast | 4.2% | 4.2% | 4.2% | |
| Cervical | 1.4% | 1.4% | 1.4% | |
| Colorectal | 7.4% | 7.4% | 7.5% | |
| Esophageal | 7.0% | 7.0% | 7.1% | |
| Liver | 10.0% | 9.8% | 10.6% | <0.001 |
| Lung | 38.8% | 39.5% | 37.1% | |
| Pancreatic | 3.5% | 3.3% | 4.2% | |
| Prostate | 1.0% | 1.0% | 0.9% | |
| Stomach | 13.2% | 13.1% | 13.6% | |
| Others | 12.4% | 12.3% | 12.9% | |
| **Aggressive Care** | | | | |
| Chemotherapy in the last 14 days | 5.8% | - | - | |
| Multiple admissions in the last 30 days | 23.0% | - | - | |
| ICU in the last 30 days | 1.9% | - | - | |
| Any one of the above | 26.7% | - | - | |

**Table S9 Modified Poisson Regression Analysis Predicting Aggressive Care (95% CI) (Patients with Metastatic Cancer)**

| Characteristics | | Chemotherapy | Multiple Admissions | ICU | Any |
|---|---|---|---|---|---|
| **Sex (ref: Male)** | Female | 0.92 (0.88 - 0.95)*** | 0.97 (0.95 - 0.98)*** | 0.97 (0.91 - 1.03) | 0.96 (0.95 - 0.98)*** |
| **Age Group (ref: 18-45)** | 46-60 | 0.83 (0.78 - 0.88)*** | 0.80 (0.78 - 0.82)*** | 0.68 (0.60 - 0.77)*** | 0.81 (0.79 - 0.83)*** |
| | 61-75 | 0.66 (0.62 - 0.70)*** | 0.61 (0.59 - 0.63)*** | 0.57 (0.51 - 0.64)*** | 0.63 (0.62 - 0.65)*** |
| | >75 | 0.36 (0.33 - 0.39)*** | 0.50 (0.48 - 0.51)*** | 0.50 (0.44 - 0.57)*** | 0.50 (0.49 - 0.52)*** |
| **Occupation and employment status (ref: Farmers)** | Public servants and retirees | 1.06 (1.01 - 1.12)* | 1.44 (1.41 - 1.47)*** | 1.49 (1.35 - 1.64)*** | 1.37 (1.34 - 1.40)*** |
| | Corporate employees | 1.00 (0.92 - 1.08) | 1.22 (1.18 - 1.26)*** | 1.19 (1.04 - 1.37)* | 1.17 (1.14 - 1.21)*** |
| | Occupation without a stable source of income | 0.89 (0.82 - 0.96)** | 1.20 (1.16 - 1.25)*** | 1.28 (1.10 - 1.49)** | 1.16 (1.12 - 1.20)*** |
| | Others | 0.86 (0.82 - 0.89)*** | 1.12 (1.10 - 1.14)*** | 1.22 (1.13 - 1.30)*** | 1.08 (1.06 - 1.10)*** |
| **Insurance Type (ref: URRBMI)** | UEBMI | 1.19 (1.14 - 1.24)*** | 1.32 (1.29 - 1.34)*** | 1.46 (1.36 - 1.58)*** | 1.30 (1.28 - 1.32)*** |
| | Uninsured | 0.94 (0.86 - 1.03) | 1.20 (1.16 - 1.25)*** | 1.82 (1.60 - 2.08)*** | 1.16 (1.12 - 1.20)*** |
| | Otherwise insured | 1.08 (1.02 - 1.16)** | 1.11 (1.08 - 1.15)*** | 1.01 (0.89 - 1.15) | 1.10 (1.07 - 1.13)*** |
| **Year of Death** | Per year | 1.01 (1.00 - 1.02)*** | 0.98 (0.98 – 0.99)*** | 1.19 (1.17 - 1.21)*** | 0.99 (0.99 - 1.00)*** |
| **Hospital (ref: Secondary Hospital)** | Tertiary Hospital | 1.03 (1.00 - 1.06) | 0.79 (0.78 - 0.80)*** | 0.96 (0.91 - 1.02) | 0.83 (0.82 - 0.84)*** |
| **Cancer type (ref:Liver)** | Brain | 1.28 (1.07 - 1.53)** | 0.70 (0.64 - 0.76)*** | 1.95 (1.48 - 2.57)*** | 0.77 (0.71 - 0.83)*** |
| | Breast | 1.98 (1.81 - 2.17)*** | 0.98 (0.94 - 1.02) | 1.61 (1.35 - 1.92)*** | 1.06 (1.02 - 1.10)** |
| | Cervical | 1.80 (1.57 - 2.06)*** | 1.00 (0.94 - 1.06) | 1.81 (1.39 - 2.34)*** | 1.08 (1.02 - 1.15)** |
| | Colorectal | 1.54 (1.42 - 1.67)*** | 1.05 (1.02 - 1.09)** | 1.63 (1.41 - 1.88)*** | 1.08 (1.05 - 1.11)*** |
| | Esophageal | 1.73 (1.59 - 1.88)*** | 1.15 (1.12 - 1.19)*** | 2.26 (1.97 - 2.59)*** | 1.19 (1.16 - 1.23)*** |
| | Lung | 1.75 (1.64 - 1.87)*** | 1.02 (1.00 - 1.04) | 2.11 (1.89 - 2.35)*** | 1.08 (1.06 - 1.10)*** |
| | Pancreatic | 1.47 (1.33 - 1.63)*** | 1.15 (1.11 - 1.19)*** | 1.03 (0.84 - 1.26) | 1.17 (1.13 - 1.21)*** |
| | Prostate | 0.94 (0.75 - 1.18) | 0.97 (0.90 - 1.04) | 1.60 (1.19 - 2.14)** | 0.99 (0.93 - 1.06) |
| | Stomach | 1.75 (1.63 - 1.89)*** | 1.12 (1.09 - 1.15)*** | 1.23 (1.07 - 1.40)** | 1.15 (1.12 - 1.18)*** |
| | Other | 1.78 (1.65 - 1.91)*** | 1.10 (1.07 - 1.13)*** | 1.77 (1.56 - 2.01)*** | 1.14 (1.11 - 1.17)*** |
| **Non-cancer CCI* (ref:>=2)** | 0 | 0.81 (0.78 - 0.84)*** | 0.49 (0.48 - 0.50)*** | 0.31 (0.29 - 0.33)*** | 0.53 (0.52 - 0.54)*** |
| | 1 | 1.05 (1.01 - 1.09)** | 0.76 (0.75 - 0.77)*** | 0.67 (0.63 - 0.71)*** | 0.79 (0.78 - 0.80)*** |

* p< 0.1, ** p< 0.05, *** p< 0.01

Abbreviation: CCI=Charlson Comorbidity Index
